# A Transformer‐Based Framework With Data Augmentation for Robust Seizure Detection Across Invasive and Noninvasive Neural Recordings

**DOI:** 10.1111/cns.70584

**Published:** 2025-09-04

**Authors:** Yue Yuan, Junyang Zhang, Chen Wang, Hao Yan, Xiangyu Ye, Wenjun Ruan, Xinzhuo Teng, Zheshan Guo, Zhaoxiang Wang

**Affiliations:** ^1^ Research Center for Life Sciences Computing Zhejiang Lab Hangzhou Zhejiang China; ^2^ State Key Laboratory of Digital Medical Engineering, School of Biomedical Engineering Hainan University Haikou Hainan China; ^3^ Key Lab of Biomedical Engineering for Ministry of Education, College of Biomedical Engineering and Instrument Science Zhejiang University Hangzhou Zhejiang China; ^4^ The Key Laboratory of Biomedical Engineering of Hainan Province, School of Biomedical Engineering Hainan University Haikou Hainan China; ^5^ Sanya Research Institute of Hainan University Sanya Hainan China

**Keywords:** cross‐modal adaptation, data augmentation, epilepsy detection, neurophysiological signals, transformer architecture

## Abstract

**Aims:**

Epilepsy affects more than 50 million peolple worldwide and requires reliable seizure detection systems to mitigate risks associated with unpredictable seizures. Existing machine learning frameworks are limited in generalizability, signal fidelity, and clinical translation, particularly when bridging invasive and non‐invasive modalities. This study aims to develop a robust and generalizable seizure detection model capable of supporting cross‐modal applicability.

**Methods:**

We proposed a Transformer‐based seizure detection framework designed for end‐to‐end analysis of raw neurophysiological signals. To address class imbalance and temporal variability, three data augmentation strategies: sequential sampling, random contiguous sampling, and random non‐contiguous sampling, were implemented. A channel‐agnostic attention mechanism was incorporated to ensure robustness across heterogeneous electrode configurations.

**Results:**

The framework achieved > 99% accuracy in detecting diverse seizure patterns from rat hippocampal recordings (CA1/CA3) and maintained strong performance across different epilepsy models (PTX‐ and 4‐AP‐induced seizures). It also demonstrated resilience under reduced‐channel configurations (F1‐score: 98.7% with 2 channels). In human electroencephalography (EEG) validation, the model achieved a recall of 99.1% and an overall accuracy of 90.4%, despite the inherent limitations of EEG in resolving high‐frequency oscillations and its susceptibility to artifacts.

**Conclusion:**

By eliminating manual feature engineering and enabling robust cross‐modal adaptation, this framework bridges invasive experimental research and non‐invasive clinical practice. Its efficiency and scalability support potential applications in real‐time seizure monitoring and closed‐loop neuromodulation systems. Future work will focus on integration with hemodynamic biomarkers, validation in chronic epilepsy models, and optimization for wearable and real‐time deployment.

## Graphical abstract

We present a Transformer‐based seizure detection framework capable of processing raw neurophysiological signals in an end‐to‐end manner. The model achieved > 99% accuracy in detecting seizures from rat recordings and 90.4% in human EEG, demonstrating strong cross‐modal adaptability. Channel‐agnostic attention enables robust performance with fewer channels (98.7% F1‐score with 2 channels).

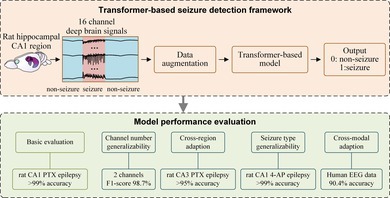

## 1 Introduction

Epilepsy is a chronic neurological disorder characterized by recurrent, unprovoked seizures resulting from abnormal excessive or synchronized neuronal firing [1, 2]. Affecting approximately 50 million individuals worldwide, epilepsy is among the most prevalent neurological conditions, with an estimated 2 million new cases reported annually [3, 4, 5]. Clinically, seizures may manifest as focal motor impairments, generalized convulsions, or transient loss of consciousness [2, 6]. These paroxysmal events typically have a sudden onset and short duration (ranging from seconds to minutes), often occurring without warning [1, 7]. These features underscore the urgent need for accurate, real‐time seizure detection methods to reduce the risks associated with unpredictable seizure onset.

Electroencephalography (EEG) remains a cornerstone for seizure detection and quantification in both clinical and research contexts [8, 9]. However, conventional diagnostic workflows relying on visual EEG interpretation by neurologists are limited by subjectivity, inefficiency in processing prolonged recordings, and susceptibility to noise artifacts [10, 11, 12]. To address these challenges, machine learning (ML) and deep learning (DL) have been explored as promising approaches for automating seizure detection [9]. Despite encouraging progress, three major challenges persist [9, 13]. First, many ML methods require manual feature extraction, while DL models often depend on preprocessing steps (e.g., spectral filtering), limiting their capacity for true end‐to‐end applicability [6, 14, 15, 16]. Second, current frameworks exhibit insufficient generalizability across seizure types, interindividual variability, and diverse EEG configurations [8, 12, 17, 18]. Third, EEG inherently suffers from low spatial resolution, attenuation of high‐frequency oscillations, and poor sensitivity to small epileptiform discharges originating from deep brain structures [6, 17, 19, 20]. These challenges highlight the need for a robust, generalizable, and fully end‐to‐end seizure detection framework.

The Transformer architecture has shown exceptional performance in time series analysis, computer vision, and computational neuroscience tasks [21, 22]. Recent research has extended its application to epilepsy‐related EEG classification with promising results [23, 24]. However, Transformer‐based models also face limitations due to EEG's baseline noise and vulnerability to high‐frequency interference [25]. In contrast, deep brain recordings from epileptogenic regions, such as the hippocampus, offer high‐fidelity spatiotemporal insights into seizure dynamics, providing an information‐rich dataset [15, 26, 27]. Yet, these experimental datasets are often constrained by limited seizure events and a significant class imbalance between seizure (positive) and non‐seizure (negative) samples. To address this, we implemented three data augmentation strategies: sequential sampling, random contiguous sampling, and random non‐contiguous sampling, to enhance seizure pattern diversity and improve model training robustness.

Building on these augmented datasets, we proposed a Transformer‐based seizure detection framework featuring four key capabilities: (1) end‐to‐end processing of raw signals without preprocessing, (2) configuration‐robust performance across heterogeneous electrode arrays and brain regions via channel‐agnostic attention mechanisms, (3) generalizability validated across both picrotoxin (PTX)‐ and 4‐aminopyridine (4‐AP)‐induced seizure models, and (4) cross‐modal adaptability to human EEG through signal upsampling. By addressing critical challenges in generalizability, signal fidelity, and clinical translation, this work contributes to the advancement of scalable, intelligent systems for closed‐loop neuromodulation and mechanistic epilepsy research.

## 2 Materials and Methods

### 2.1 Data Acquisition

To evaluate the performance of the proposed model, we employed long‐term, continuous, multi‐channel datasets. Training data were obtained from acute animal experiments, while test data comprised both animal recordings and publicly available EEG datasets from the TUEP Corpus.

#### 2.1.1 Animal Data

**FIGURE 1:**
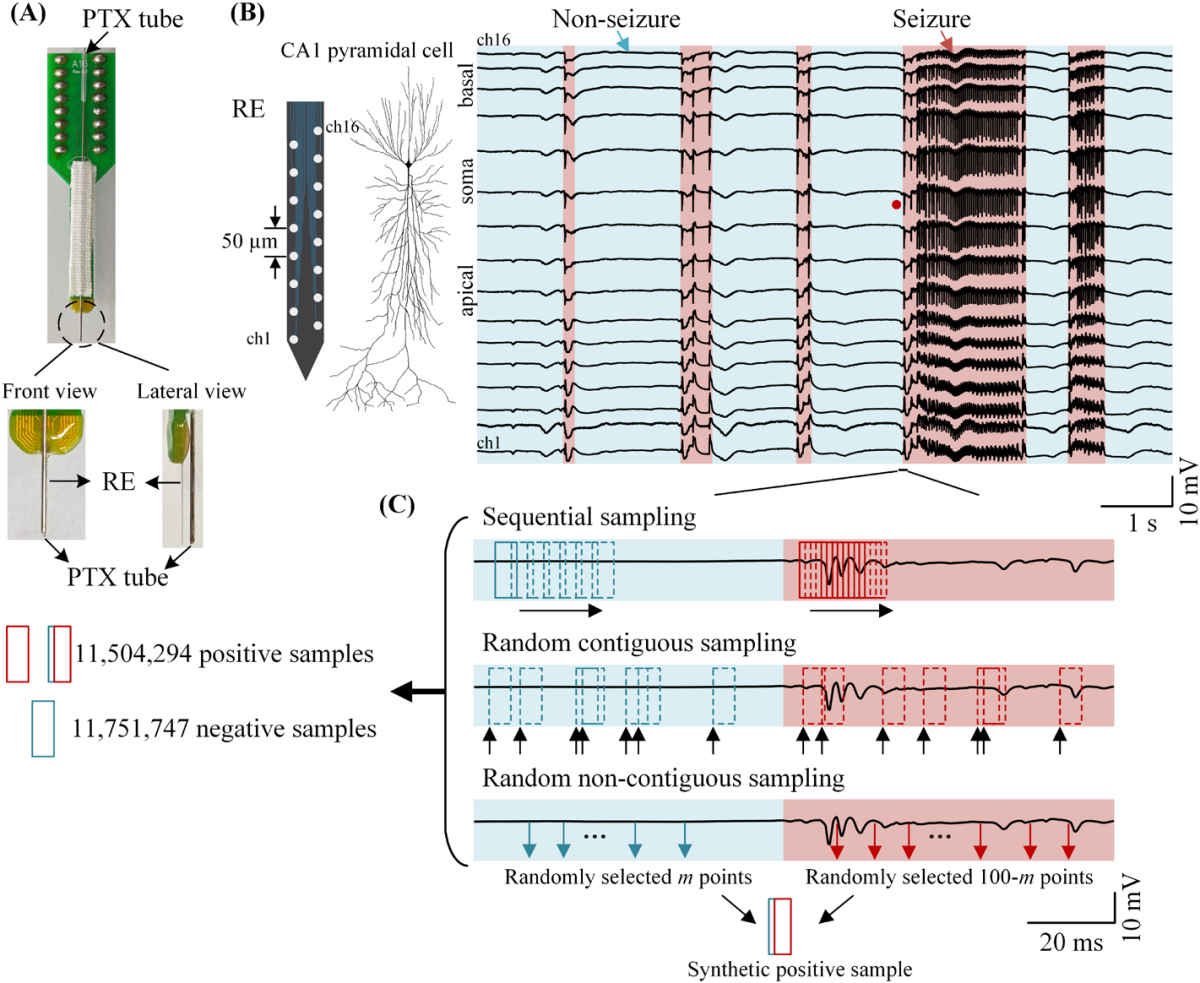
Schematic of data acquisition and data augmentation for training local field potential data. (A) The spatial relationship between the recording electrode (RE) and the PTX injection tube. In the front view, the RE and PTX tube are aligned vertically; in the lateral view, the PTX tube is positioned ~1 mm away from the RE. (B) Representative 16‐channel local field potential (LFP) recordings from hippocampal CA1 region, aligned along the basal dendrites, soma, and apical dendrites of a pyramidal neuron. The signals include both non‐seizure (blue shaded areas) and epileptiform discharge periods (red shaded areas). The signal from the soma layer (marked by a red dot) is expanded in (C). (C) Illustration of data augmentation strategies. Each sample window spans 5 ms (100 data points at 20 kHz sampling rate), with blue boxes indicating negative samples (non‐seizure) and red boxes as well as “blue + red” combinations indicating positive samples. In sequential sampling, sliding sample windows traverse the long‐duration LFP signals with a fixed stride (e.g., 25 points shown here), starting from the very first data point of each segment. In random contiguous sampling, each window is independently and randomly selected along the time axis, with the total amount of windows predefined. In random non‐contiguous sampling, synthetic positive samples are constructed by randomly selecting *m* data points (*m* < 40) from nonseizure segments and (100 − *m*) data points from seizure segments.

Experimental data were collected from 25 adult male Sprague–Dawley rats (250–300 g). All procedures were approved by the Laboratory Animal Welfare and Ethics Committee of Zhejiang University (ZJU20210108). During each experiment, the rat was anesthetized with urethane (1.25 g/kg, i.p.) and secured in a stereotaxic apparatus (Stoelting Co., USA). A portion of the skull was removed to implant a 16‐channel recording array (NeuroNexus Technologies, USA) into the left hippocampal CA1 region (AP, −3.5 mm; ML, 2.7 mm; DV, ~2.5 mm). A stainless‐steel guide cannula (outer diameter: 0.3 mm) was positioned adjacent to the recording electrode to allow subsequent drug injections (Figure 1A).

The recording electrode (A1 × 16‐Poly2‐5 mm‐50s‐177) comprised 16 contacts arranged in two columns, with 50 μm spacing, enabling simultaneous recordings from the apical dendrites, soma, and basal dendrites of the hippocampal CA1 region (Figure 1B). Extracellular potentials recorded by the electrode were amplified 100‐fold and filtered between 0.3 and 5000 Hz using a 16‐channel amplifier (Model 3600, A‐M Systems Inc., USA). The filtered signals were then digitized at 20 kHz with 16‐bit resolution via a PowerLab data acquisition system (PL3516, AD Instruments Inc., Australia).

An acute seizure model was induced by microinjecting picrotoxin (PTX, 4 mM), a GABA_a_ receptor antagonist, through the cannula targeting the dorsal hippocampus (AP, −3.5 mm; ML, 2.0 mm; DV, ~2.0 mm). Injections (0.5 μL) were administered at 1–5 min intervals using a custom injector and a 25 μL microsyringe, with a total injection volume of 3–5 μL per rat, until stable epileptiform activities emerged.

To further evaluate the model's generalizability, we included two additional datasets: (1) PTX‐induced seizures recorded from the hippocampal CA3 region (AP, −3.1 mm; ML, 3.0 mm; DV, ~3.0 mm); and (2) seizures induced by 4‐aminopyridine (4‐AP, 40 mM) in the CA1 region [28].

#### 2.1.2 Human Data

Scalp EEG recordings from the TUEP Corpus (version v2.0.1), a subset of the TUH EEG Data Corpus (https://isip.piconepress.com/projects/nedc/html/tuh_eeg/), were used to evaluate the model's performance on human seizure detection [29]. To assess generalization across sampling rates, we selected multiple EEG sessions from the epilepsy category with varying acquisition frequencies. All EEG traces were visually reviewed by an experienced epileptologist to identify seizure onsets and offsets and to exclude persistently artifact‐contaminated channels. Details of the selected datasets are summarized in Table 1.

**TABLE 1:** Details of the TUEP Corpus EEG data used in this study.

EEG file name	Sample rate (Hz)	Selected period	Selected channels	Number of seizures
aaaaaanr_s006_t001	256	46:40–49:00	ch 2–21	136
aaaaaanr_s007_t003	256	0:00–1:46	ch 4–19	43
aaaaalqm_s004_t000	1000	4:50–7:10	ch 3–22	10
aaaaalzg_s026_t000	256	2:20–5:20	ch 2–21	13
aaaaamde_s005_t001	250	11:40–14:40	ch 4–23	17

### 2.2 Dataset Preparation

Continuous 16‐channel neural recordings were manually annotated by experienced electrophysiologists and categorized into seizure and non‐seizure periods (highlighted in red and blue, respectively in Figure 1B ). Non‐seizure data included both baseline (pre‐injection) and inter‐ictal periods, ensuring a comprehensive representation of non‐pathological activity. In total, 482 seizure segments (duration: 0.007–5.5 s) and 89 non‐seizure segments (duration: 1–640 s) were extracted from the rat recordings.

To address the pronounced imbalance between transient seizures and prolonged non‐seizure periods, three data augmentation strategies were employed (Figure 1C):

1. Sequential sampling. A fixed‐length sliding window was used to traverse the entire dataset. For seizure segments, the stride was 1 data point, for non‐seizure segments, the stride was 50 data points, generating temporally aligned positive and negative samples.
2. Random contiguous sampling. A predefined number of windows were randomly selected from both seizure and non‐seizure periods to enhance temporal variability and improve model generalization.
3. Random non‐contiguous sampling. Synthetic training samples were generated by randomly combining 100 data points per channel: *m* points (*m* < 40) from non‐seizure segments and (100 − *m*) points from seizure segments. This hybrid sampling approach was designed to improve the model's sensitivity to transitional patterns between seizure and non‐seizure states.

In all augmentation strategies, a window size of 5‐ms (100 data points at 20 kHz) was selected to capture the temporal features of seizure‐related waveforms. After augmentation, the final dataset comprised 11,751,747 negative and 11,504,294 positive samples, each formatted as a 16 × 100 matrix (channels × temporal features).

### 2.3 Proposed Method

**FIGURE 2:**
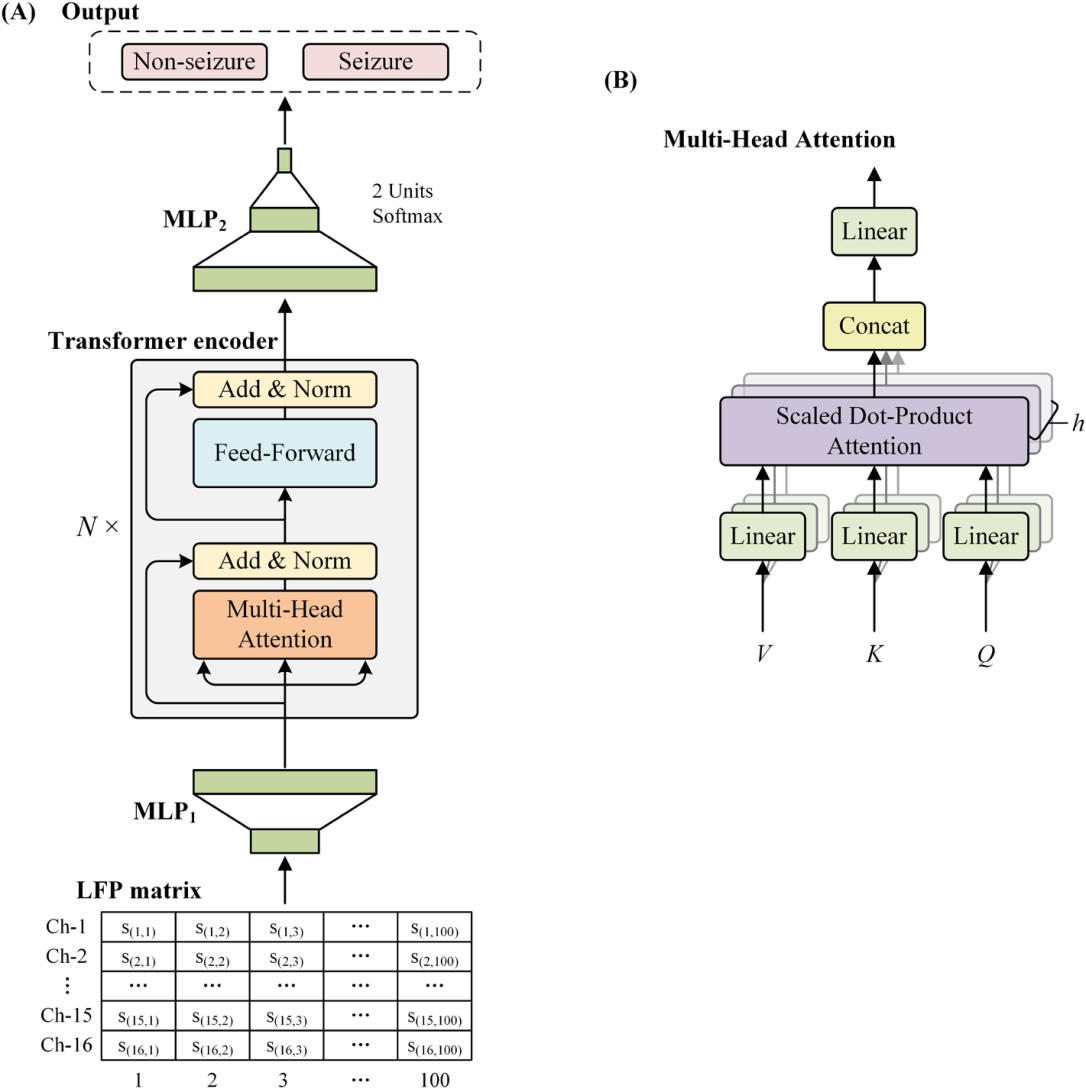
Architecture and flow diagram of the proposed method. (A) Overall workflow of the proposed Transformer‐based model. (B) Structure of the Multi‐Head Attention mechanism used in the Transformer encoder.

The Transformer architecture has demonstrated strong performance across time series classification, computer vision, and predictive modeling tasks [21, 22]. Recent studies have further validated its applicability in epilepsy‐related EEG classification [23, 24]. Building upon these advances, we designed a Transformer‐based neural network optimized for sequential neural signal modeling. The proposed architecture consists of four main components: an input projection layer (a fully connected neural network), eight Transformer encoder layers, and two output projection layers (also fully connected) (Figure 2A).

#### 2.3.1 Input Projection Layer

To ensure compatibility with the multi‐head Transformer encoder, the original input, formatted as a 16 × 100 matrix, was projected via a fully connected mapping layer (MLP_1_) into a higher‐dimensional shape of 16 × 1024, that is,

(1) $$X_{16\times 1024}={\mathrm{MLP}}_{1}\left(X_{16\times 100}\right)$$

#### 2.3.2 Transformer Encoder Layers

The encoder comprises *N* = 8 identical Transformer layers. Each layer integrates a multi‐head self‐attention mechanism and a position‐wise fully connected feed‐forward network. Residual connections and layer normalization are applied after each sub‐layer [30, 31]. The output of each sublayer is

(2) $$\text{LayerNorm}\left(x+\text{Sublayer}\left(x\right)\right)$$

and generating outputs with a fixed dimensionality of *d* = 1024.

#### 2.3.3 Attention Mechanism

An attention function maps a query (*Q*), key (*K*), and value (*V*) to an output (Figure 2B). The output is a weighted sum of *V*, where the weights are determined by a compatibility function of the *Q* with the corresponding *K*. The dimensions of *Q* and *K* are denoted as *d*_*k*_, and of *V* as *d*_*v*_. In our model, we set *d*_*k*_ = *d*_*v*_ = *d*_*model*_ /8 = 128.

The output matrix is computed as:

(3) $$\text{Attention}\left(Q,K,V\right)=\text{softmax}\left(\frac{{\mathrm{QK}}^{T}}{\sqrt{d_{k}}}\right)V$$

The multi‐head attention mechanism further improves the self‐attention layer, expands the ability of the model to focus on different positions, and gives multiple “representation subspaces” in the attention layer, which is expressed as:
(4)

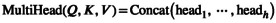

Each head is computed as:

(5) $${\text{head}}_{i}=\text{Attention}\left({\mathrm{QW}}_{i}^{Q}{\mathrm{KW}}_{i}^{K},{\mathrm{VW}}_{i}^{V}\right)$$

where *h* is the number of attention heads, $W_{i}^{Q}$, $W_{i}^{K}$, $W_{i}^{V}$ are the learned projection matrices.

In this work, we set *h* = 8 attention heads. In addition to attention sublayers, each Transformer encoder layer contains a fully connected feed‐forward network, which is applied to each position separately and identically. This consists of two linear transformations with a ReLU activation in between. The dimension of the feed‐forward network is *d*_*ff*_ = 1024. Thus, the output of the Transformer Layer is:

(6) $$Y_{16\times 1024}=8\times \text{TransformersLayers}\left(X_{16\times 1024}\right)$$

#### 2.3.4 Output Projection Layer

After passing through eight layers of Transformer encoders, the output dimension remains 16 × 1024. To reduce model complexity and mitigate overfitting, we introduce MLP_2_, which contains two fully connected layers to reduce the output dimension. Namely:

(7) $$\text{output}={\mathrm{MLP}}_{2}\left(Y_{16\times 1024}\right)$$

(8) $$\text{score}=\text{softmax}\left(\text{output}\left[0,:\right]\right)$$

Finally, the output of the entire model is scored through the *softmax* function to give the prediction result of the corresponding input matrix:

(9) $$\text{Softmax}\left(z_{i}\right)=\frac{\exp\left(z_{i}\right)}{\sum_{j}\exp\left(z_{j}\right)}$$

### 2.4 Hardware and Training

Model training was conducted on a high‐performance computing server equipped with an Intel Xeon Gold 5218R CPU (80 cores, 2.10 GHz), 512 GB RAM, and an NVIDIA RTX A6000 GPU (48 GB VRAM). The training pipeline was implemented in PyTorch (v 2.3.1) using CUDA (v 12.5). The hyperparameters were set as follows: a learning rate of 1 × 10^−5^, 100 training epochs, a batch size of 2048, 8 attention heads, and 8 Transformer encoder layers. The Adam optimizer was used with default settings. The model was trained using cross‐entropy loss until convergence on the validation set. The final model was selected based on the highest validation accuracy achieved during training.

### 2.5 Postprocessing and Evaluation

During inference, the model was evaluated with step sizes ranging from 5 to 20 data points, corresponding to a temporal resolution of 0.25 to 1 ms. The model's output was the indices of detected epileptiform activities, allowing it to identify complete seizure activities. Consequently, the detection flags in the subsequent figures consistently appear as clusters of consecutive “1” s. For interpretability, two seizure segments were considered distinct events only if the interval between them exceeded 400 data points (equivalent to 20 ms); otherwise, they were merged into a single event. This threshold was selected based on the minimum inter‐seizure intervals in vivo.

Standard classification metrics were introduced to evaluate the performance of the proposed method, including recall (sensitivity), precision, F1‐score, and positive predict accuracy. The calculation formulae are as follows:

(10) $$\text{Recall}=\text{Sensitivity}=\frac{\mathrm{TP}}{\mathrm{TP}+\mathrm{FN}}$$

(11) $$\text{Precision}=\frac{\mathrm{TP}}{\mathrm{TP}+\mathrm{FP}}$$

(12) $$F1‐\text{score}=2\times \frac{\text{Precision}\times \text{Recall}}{\text{Precision}+\text{Recall}}$$

(13) $$\text{Accuracy}=\frac{\mathrm{TP}}{\mathrm{TP}+\mathrm{FP}+\mathrm{FN}}$$

where TP, FP, FN represent true positive, false positive, and false negative, respectively.

## 3 Results

### 3.1 Basic Evaluation of the Proposed Model

**FIGURE 3:**
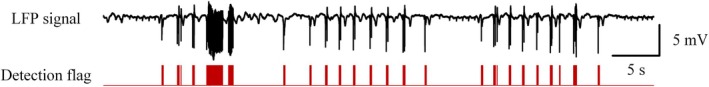
Typical example showing the detection result in rat hippocampal CA1 region in PTX epilepsy model.

We first evaluated the proposed model using neural recordings acquired from 8 rats within the same PTX‐induced seizure models used for training dataset generation. For each rat, 2‐min continuous 16‐channel recordings were analyzed, capturing both seizure and inter‐seizure activities. The model showed strong discriminative capability, accurately separating seizure firing from non‐seizure intervals (Figure 3). Quantitative evaluation results (Table 2) revealed near‐perfect performance: recall = 100%, precision = 99.4% ± 0.9%, F1‐score = 99.7% ± 0.4%, and accuracy = 99.4% ± 0.9%.

**TABLE 2:** Evaluation results in rat hippocampal CA1 region in PTX epilepsy model.

Rat	Number of seizures	Recall (%)	Precision (%)	F1‐score (%)	Accuracy (%)
1	74	100	98.7	99.3	98.7
2	158	100	97.5	98.8	97.5
3	68	100	100	100	100
4	41	100	100	100	100
5	11	100	100	100	100
6	159	100	99.4	99.7	99.4
7	92	100	100	100	100
8	47	100	100	100	100
Average	—	100	99.4 ± 0.9	99.7 ± 0.4	99.4 ± 0.9

To address heterogeneity in recording setups (e.g., channel counts and spatial configurations), we systematically evaluated the model's generalizability under different channel numbers (1, 2, 4, or 8 channels). By randomly subsampling channels (1, 2, 4, 8) from the PTX testing dataset, we observed robust seizure detection across configurations (Table 3), albeit with performance variations dependent on channel counts. Nevertheless, single‐channel configurations exhibited reduced sensitivity (recall: 85.5% ± 20.7%) compared to 8‐channel setups (100%). Conversely, higher channel numbers (e.g., 4 or 8 channels) led to more false positives, thereby leading to lower precision.

**TABLE 3:** Evaluation results in PTX epilepsy model with different channel number.

Channel number	Recall (%)	Precision (%)	F1‐score (%)	Accuracy (%)
1	85.5 ± 20.7	100	90.4 ± 15.7	85.5 ± 20.7
2	97.6 ± 5.1	100	98.7 ± 2.7	97.6 ± 5.1
4	99.9 ± 0.4	91.9 ± 14.9	95.0 ± 9.7	91.7 ± 14.8
8	100	92.3 ± 12.9	95.4 ± 8.5	92.3 ± 12.9

Epilepsy is a network‐level neurological disorder characterized by propagating pathological activity and region‐specific epileptiform patterns [32, 33], so we next evaluated the model's ability to generalize across different brain regions.

### 3.2 Evaluation of the Proposed Model in Different Brain Regions and Epilepsy Models

In the PTX‐induced acute epilepsy models, seizure activities recorded from the hippocampal CA3 region exhibited distinct spatiotemporal dynamics compared to those in the CA1 region [27]. To evaluate the model's cross‐region generalizability, we analyzed 2‐min continuous 16‐channel recordings from the rat hippocampal CA3 region. The model maintained high performance, with a recall of 99.7% ± 0.6%, a precision of 95.8% ± 3.4%, an F1‐score of 97.7% ± 1.9%, and an accuracy of 95.6% ± 3.7% (Table 4), indicating strong adaptability to diverse epileptogenic zones.

**TABLE 4:** Evaluation results in rat hippocampal CA3 region in PTX epilepsy model.

Rat	Number of seizures	Channel number	Recall (%)	Precision (%)	F1‐score (%)	Accuracy (%)
1	28	8	100	100	100	100
2	14	8	100	93.3	96.6	93.3
3	45	4	100	93.8	96.8	93.8
4	71	8	98.6	92.1	95.2	90.9
5	154	8	100	100	100	100
Average	—	—	99.7 ± 0.6	95.8 ± 3.4	97.7 ± 1.9	95.6 ± 3.7

Seizures induced by different pharmacological agents also display distinct electrophysiological patterns. For instance, 4‐AP‐induced epileptiform activities typically manifest as high‐frequency spiking followed by pseudo‐periodic spikes, whereas PTX‐induced discharges are characterized by large‐amplitude bursts. To evaluate the model's generalizability across various seizure types, we further applied it to neural signals obtained from the 4‐AP‐induced epilepsy models. The experimental procedures used to establish the 4‐AP acute epilepsy models were consistent with those described in Section 2.1.1.

**FIGURE 4:**
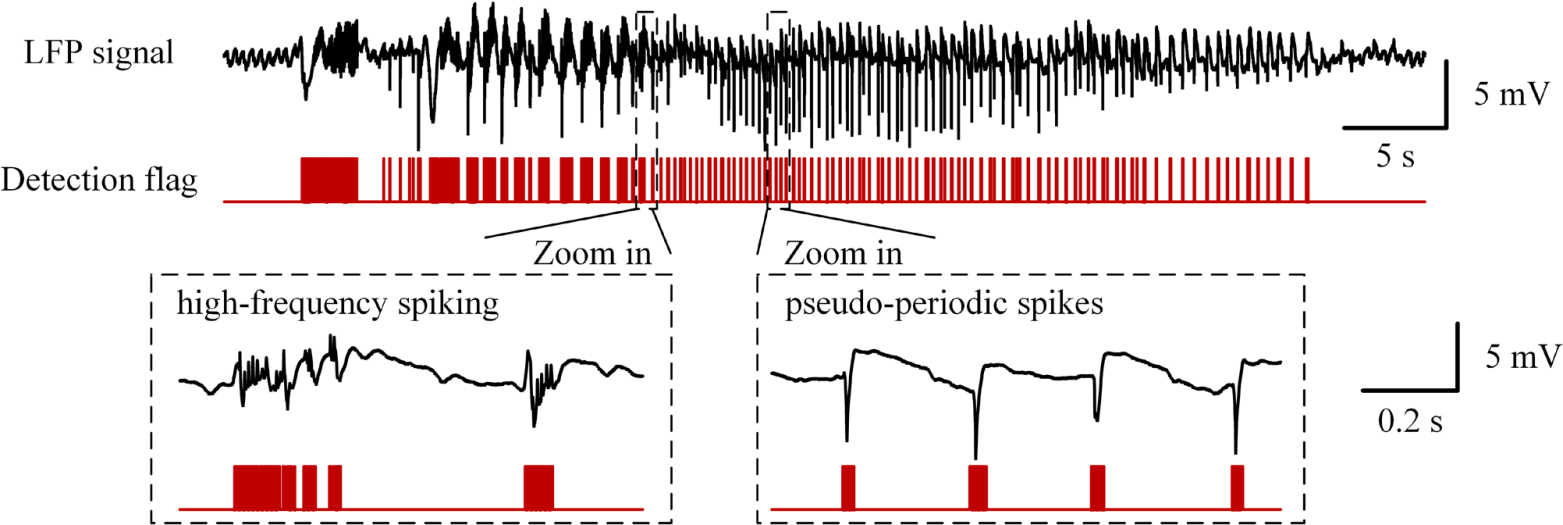
Typical example showing the model's generalizability in rat hippocampal CA1 region in 4‐AP epilepsy model.

Figure 4 illustrates a representative detection result from the 4‐AP dataset, demonstrating the model's precise identification of both high‐frequency spiking and pseudo‐periodic spikes. Quantitative evaluation (Table 5) further validated the framework's robustness, achieving near‐perfect performance metrics: recall = 99.9% ± 0.3%, precision = 99.9% ± 0.2%, F1‐score = 99.9% ± 0.1%, and accuracy = 99.8% ± 0.3%.

**TABLE 5:** Evaluation results in rat hippocampal CA1 region in 4‐AP epilepsy model.

Rat	Number of seizures	Recall (%)	Precision (%)	F1‐score (%)	Accuracy (%)
1	74	100	100	100	100
2	146	99.3	100	99.7	99.3
3	67	100	100	100	100
4	15	100	100	100	100
5	219	100	99.5	99.8	99.5
6	143	100	100	100	100
Average	—	99.9 ± 0.3	99.9 ± 0.2	99.9 ± 0.1	99.8 ± 0.3

These results demonstrated the model's robustness in detecting diverse seizure patterns across experimental conditions using deep brain neural signals. To evaluate its translational potential in clinical practice, where scalp EEG remains the primary diagnostic modality, we further validated the framework's generalizability on human EEG signals for seizure detection.

### 3.3 Evaluation of the Proposed Model in Human EEG Data

To assess performance in clinical settings, we utilized scalp EEG data from the TUEP Corpus, a subset of the TUH EEG Data Corpus. Since the original EEG recordings were sampled at relatively low rates (e.g., 256 Hz, as detailed in Table 1), we first upsampled the signals to 25.6 kHz (for 256 Hz data) or 20 kHz (for 1000 and 250 Hz data) using linear interpolation to match the temporal resolution of invasive deep brain recordings used during training. The upsampled signals were then normalized by dividing each signal by a factor of 500.

**FIGURE 5:**
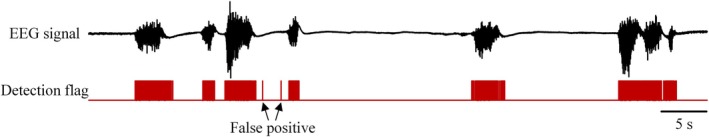
Typical example showing the model's performance in Human EEG data.

Figure 5 illustrates a representative example of the model's performance on human EEG data, demonstrating successful detection of most seizure events despite sporadic false positives. Quantitative evaluation (Table 6) further validated the framework's efficacy, achieving a recall of 99.1% ± 1.9%, precision of 91.3% ± 4.7%, F1‐score of 94.9% ± 2.1%, and accuracy of 90.4% ± 3.8%.

**TABLE 6:** Evaluation results of Human EEG data in TUEP Corpus.

Patient ID	Number of seizures	Recall (%)	Precision (%)	F1‐score (%)	Accuracy (%)
aaaaaanr_s006_t001	136	100	93.2	96.5	93.2
aaaaaanr_s007_t003	43	95.3	97.6	96.5	93.2
aaaaalqm_s004_t000	10	100	83.3	90.9	83.3
aaaaalzg_s026_t000	13	100	92.9	96.3	92.9
aaaaamde_s005_t001	17	100	89.5	94.4	89.5
Average	—	99.1 ± 1.9	91.3 ± 4.7	94.9 ± 2.1	90.4 ± 3.8

## 4 Discussion

The proposed Transformer‐based framework addresses three critical challenges in automated seizure detection: (1) configuration‐robust generalizability across recording setups and epilepsy models, (2) end‐to‐end processing of raw neurophysiological signals, and (3) cross‐modal adaptation to clinical EEG. Below, we critically analyze the physiological and technological implications of these advancements, identify methodological limitations, and propose future directions.

### 4.1 Methodological Advancements and Physiological Insights

Deep brain recordings from epileptogenic zones (e.g., hippocampal CA1) provide high‐fidelity spatiotemporal characterization of ictal dynamics, capturing seizure propagation at the millisecond scale [15, 26, 34]. The proposed data augmentation framework addressed three limitations in experimental seizure datasets: limited seizure events, severe class imbalance, and restricted temporal variability [35]. The sequential sampling strategy preserves the intrinsic temporal continuity of seizures, while random contiguous and non‐contiguous sampling techniques mimic transitional seizure dynamics by strategically blending seizure and non‐seizure segments. This approach not only effectively alleviates class imbalance but also compels the model to recognize subtle transitional features between physiological and pathological brain states.

The Transformer's channel‐agnostic self‐attention mechanism dynamically weights to inter‐electrode relationships, allowing robust extraction of spatiotemporal seizure biomarkers from augmented datasets [22, 36]. Consequently, the proposed Transformer‐based framework achieved > 99% classification accuracy across both PTX‐ and 4‐AP‐induced seizure models in the rat hippocampal CA1 region, despite the distinct pathophysiological mechanisms underlying PTX‐induced epileptiform bursts and 4‐AP‐driven high‐frequency spiking. Notably, the model generalized to PTX‐induced CA3 recordings (> 95% accuracy) without retraining, demonstrating cross‐region adaptability critical for clinical environments with variable electrode placements. In contrast to invasive recordings, EEG signals exhibit inherent limitations: high‐frequency oscillations and low‐amplitude epileptiform patterns are attenuated by signal transmission through extracranial tissues (skull, meninges, and scalp), leading to incomplete seizure dynamics representation [37, 38].

Moreover, the framework maintained clinically relevant performance (F1‐score: 98.7%) with only 2 channels, which is a critical capability given intraoperative electrode placement constraints and patient‐specific anatomical variability. This channel‐agnostic design, inherent to the Transformer's self‐attention mechanism, adaptively weights input channels by quantifying inter‐electrode signal relationships—effectively emulating clinicians' capacity to prioritize salient neurophysiological patterns during diagnostic interpretation. Such dynamic channel prioritization, absent in conventional static architectures, ensures reliable operation across variable recording setups while preserving sensitivity to high‐frequency oscillations and low‐amplitude epileptiform biomarkers.

### 4.2 Bridging Invasive and Noninvasive Modalities

Although the model was trained exclusively on invasive deep brain recordings, its cross‐modal adaptability to human EEG (90.4% accuracy) underscores its translational potential. By upsampling EEG signals to match deep brain recording characteristics, we partially mitigated the resolution gap between modalities. Nevertheless, the performance gap between rat and human datasets (99.4% vs. 90.4% accuracy) can be attributed to two factors: (1) EEG's inherent inability to resolve high‐frequency components [38, 39, 40, 41], and (2) EEG's lower signal‐to‐noise ratio and increased noise from muscle artifacts and non‐epileptiform transients in clinical recordings [40, 42]. Moreover, the linear interpolation upsampling strategy used here may introduce artificial high‐frequency components, potentially distorting signal characteristics. Future work is needed to incorporate artifact suppression techniques and alternative upsampling methods (such as Sinc or polynomial interpolation) to enhance robustness. Additionally, the human dataset's small sample size (5 patients) limits statistical power. Expanding validation to larger cohorts, including intracranial EEG (iEEG) from drug‐resistant epilepsy patients, would strengthen clinical relevance [43].

### 4.3 Implications and Practical Considerations

The proposed framework eliminates the need for manual feature extraction or signal preprocessing (e.g., spectral filtering), enabling truly end‐to‐end seizure detection. Unlike conventional ML/DL models that depend heavily on handcrafted features or preprocessing pipelines [9, 18, 20, 44], our approach directly processes raw deep brain signals, eliminating preprocessing biases and preserving high‐frequency oscillations and weak epileptiform features. Such capability is essential for capturing the rapid spatiotemporal dynamics of seizures, thereby significantly improving both detection accuracy and cross‐domain generalizability. The framework's end‐to‐end design eliminates preprocessing steps, reducing deployment barriers in resource‐limited settings. Its computational efficiency (trained on a single GPU) further supports real‐time applications, such as closed‐loop neuromodulation systems. However, three practical challenges merit further consideration: (1) While the 5‐ms detection window captures brief epileptiform events, real‐time deployment demands hardware‐specific optimizations to minimize inference delays. (2) Acute rodent models lack the neuroplastic changes and recurrent seizures seen in chronic epilepsy. Future validation on long‐term recordings (e.g., in vivo models of temporal lobe epilepsy) is essential. (3) Although attention maps generated by the Transformer could theoretically highlight seizure‐related features, their clinical interpretability has not yet been established. Future work should include visualizing attention weights to link model predictions with established electrophysiological biomarkers.

In addition, the integrity of cerebral circulation is increasingly recognized as a modulator of epilepsy, with seizure events triggering transient hemodynamic changes that may exacerbate neuronal hyperexcitability [45, 46]. Although our framework focuses on electrophysiological biomarkers, emerging multimodal neuroimaging technologies, such as concurrent EEG and functional near‐infrared spectroscopy (fNIRS), offer opportunities to integrate cerebral blood flow dynamics into seizure detection paradigms [47, 48]. It could be fused with our Transformer architecture to create a multiscale seizure monitoring framework. However, translating these multimodal approaches into clinical practice faces two key challenges: (1) Electrophysiological signals (μs‐ms scale) and hemodynamic responses (seconds‐minutes scale) operate on divergent timescales. While our 5‐ms detection window is well‐suited for capturing rapid seizure dynamics, integrating slower hemodynamic features may require adaptive windowing. For instance, detecting gradual hemodynamic changes may necessitate a window of 5 s or longer [49]. (2) Current artificial intelligence models often treat modalities as independent inputs rather than interconnected systems. Further studies should explore attention mechanisms that dynamically weight electrophysiological and hemodynamic features based on disease stage—for example, prioritizing EEG spikes during seizure onset while emphasizing fNIRS hypoxia markers in postseizure periods [50].

### 4.4 Limitations and Future Directions

While the study demonstrates promising results, several limitations warranting further consideration must be acknowledged. First, the reliance on acute rat epilepsy models may not fully replicate the chronic progression of human epilepsy pathophysiology [51]. Validating the framework with chronic epilepsy datasets or human iEEG recordings could significantly enhance its clinical translational value. Second, the current EEG upsampling methodology introduces artificial high‐frequency components that might distort signal characteristics. Future efforts should consider advanced domain adaptation techniques, such as adversarial training architectures, could better preserve physiological signal integrity and improve generalization across modalities. Third, future development should prioritize two interdependent engineering objectives: (1) Creating lightweight model variants optimized for wearable devices using techniques such as neural architecture search and quantization techniques, and (2) Implementing latency‐aware optimization strategies to facilitate real‐time seizure monitoring under strict power constraints. These advancements are particularly crucial for deployment in resource‐limited clinical environments. Finally, the role of cerebral circulation in seizure termination and recurrence remains underexplored in our framework. The integration of EEG, fNIRS, and angiography data to construct multimodal datasets for epilepsy models could reveal how hemodynamic influences seizure dynamics [49, 52].

## 5 Conclusion

This study establishes a Transformer‐based framework as a robust and versatile tool for seizure detection, effectively bridging the gap between invasive research and noninvasive clinical practice. By addressing key limitations in generalizability, signal fidelity, and cross‐modal adaptation, the work lays the groundwork for next‐generation epilepsy diagnostics and closed‐loop therapeutic systems. Continued efforts to enhance interpretability, expand validation cohorts, and optimize real‐world deployment will be essential for realizing the full clinical potential of this approach.

## Ethics Statement

All animal procedures were approved by the Laboratory Animal Welfare and Ethics Committee of Zhejiang University (ZJU20210108).

## Conflicts of Interest

The authors declare no conflicts of interest.

## Data Availability

The data that support the findings of this study are openly available in GitHub at https://github.com/nudtzjy/epilepsy.
